# Atypical fibrolipomatous vascular proliferative lesion mimicking Orbital fat prolapse in the lower eyelid: A case report and literature review

**DOI:** 10.1016/j.jpra.2026.09.004

**Published:** 2026-09-12

**Authors:** Peixian Yang, Mengtian Bai, Feiyan Zhu, Jing Kong, Xuan Liao, DaQing Wang

**Affiliations:** Department of Ophthalmology,Affiliated Hospital of North Sichuan Medical College, Nanchong, Sichuan 637000, China

**Keywords:** Orbital fat prolapse, Orbital mass, Fibrolipomatous lesion, Fibrovascular proliferation, Misdiagnosis, Case report

## Abstract

Orbital fat prolapse (OFP) is a common age-related condition usually diagnosed clinically and managed with excision and septal repair. However, multiple orbital pathologies can mimic OFP and cause diagnostic pitfalls. We report a rare case of fibrolipomatous vascular proliferative lesion initially misdiagnosed as lower eyelid OFP. A 61-year-old male presented with progressive left lower eyelid drooping for one month, with clinical features consistent with OFP. Intraoperatively, a firm, mobile, pale deep orbital mass was found distinct from typical adipose tissue, and complete excision was performed. Histopathology showed benign proliferation of fibrous tissue, mature adipocytes and small thin-walled vessels, without atypia or mitosis. No recurrence was observed during follow-up. This case highlights that fibrovascular orbital lesions can masquerade as simple OFP. Surgeons should maintain vigilance for atypical pathology when intraoperative findings deviate from preoperative expectations, and histopathological confirmation is essential for definitive diagnosis.

## Introduction

This report was prepared in accordance with the SCARE 2020 guidelines.^1^

Orbital fat prolapse (OFP) is a prevalent ophthalmic condition in elderly populations, caused by herniation of intraconal orbital fat through weakened orbital septum into the subcutaneous or subconjunctival space.^2^ Diagnosis is predominantly based on clinical manifestations, palpation and slit-lamp examination, and most patients undergo direct surgical excision for aesthetic improvement or functional relief^2^

However, a broad spectrum of orbital lesions can clinically mimic OFP, including prolapsed lacrimal gland, lymphoma, dermolipoma and various mesenchymal tumors, leading to potential misdiagnosis.2], 3, 4, 5 Previous studies have documented malignant lymphoma and subconjunctival nerve sheath myxoma presenting as apparent fat prolapse, which were only confirmed by postoperative histopathology.^4^^,^^5^ Since preoperative advanced imaging is not routinely performed for typical OFP, deep-seated atypical lesions may be overlooked before surgery.

This case report was written following the CARE (CAse REport) guidelines and the SCARE 2020 consensus-based surgical case report guidelines.^1^^,^^10^ Written informed consent was obtained from the patient for publication of this report and accompanying images.The signed original informed consent form is archived at our institution and can be provided in full at any time upon the journal’s request.

## Case report

A 61-year-old male presented with a one-month history of progressive left lower eyelid drooping. The patient first noticed left lower eyelid fullness five weeks prior to presentation, which was painless and not associated with visual disturbance, diplopia or ocular motility limitation. He sought treatment mainly for aesthetic concern and to exclude pathological lesions. No history of periorbital trauma, prior ocular surgery, systemic inflammatory disease or chronic comorbidities was reported. Family history was unremarkable.

On examination, best-corrected visual acuity of the left eye was 0.8. Intraocular pressure was 15 mmHg in the right eye and 17 mmHg in the left eye. Ocular motility was full and symmetric, with no signs of proptosis. External examination showed mild laxity and inferior displacement of the left lower eyelid, with diffuse soft tissue fullness without cutaneous discoloration. Palpation along the left inferior orbital rim revealed a soft, reducible mass, consistent with subcutaneous orbital fat herniation. Anterior segment and fundoscopic examinations were unremarkable bilaterally. A preoperative diagnosis of left lower eyelid orbital fat prolapse was made.

Surgery was performed under local anesthesia. A skin incision was made along the lower eyelid marking, followed by sequential dissection of skin, fascia and orbicularis oculi muscle to expose and incise the orbital septum. Upon deep exploration of the orbital cavity, no typical mobile adipose mass was palpated through the primary incision. An auxiliary incision was then made adjacent to the nasal ala; digital compression extruded a pale, firm mass with good mobility, which was clearly distinct from the surrounding yellow orbital fat. The mass, located deep in the inferior orbit, was completely excised along with adjacent adipose tissue and sent for histopathological examination. For aesthetic symmetry, a routine lower eyelid blepharoplasty with excision of unremarkable orbital fat was also performed on the contralateral (right) side. No upper eyelid surgery was performed.The orbital septum was then repaired. Intraoperative blood loss was minimal, and no complications occurred.

**Fig. 1:**
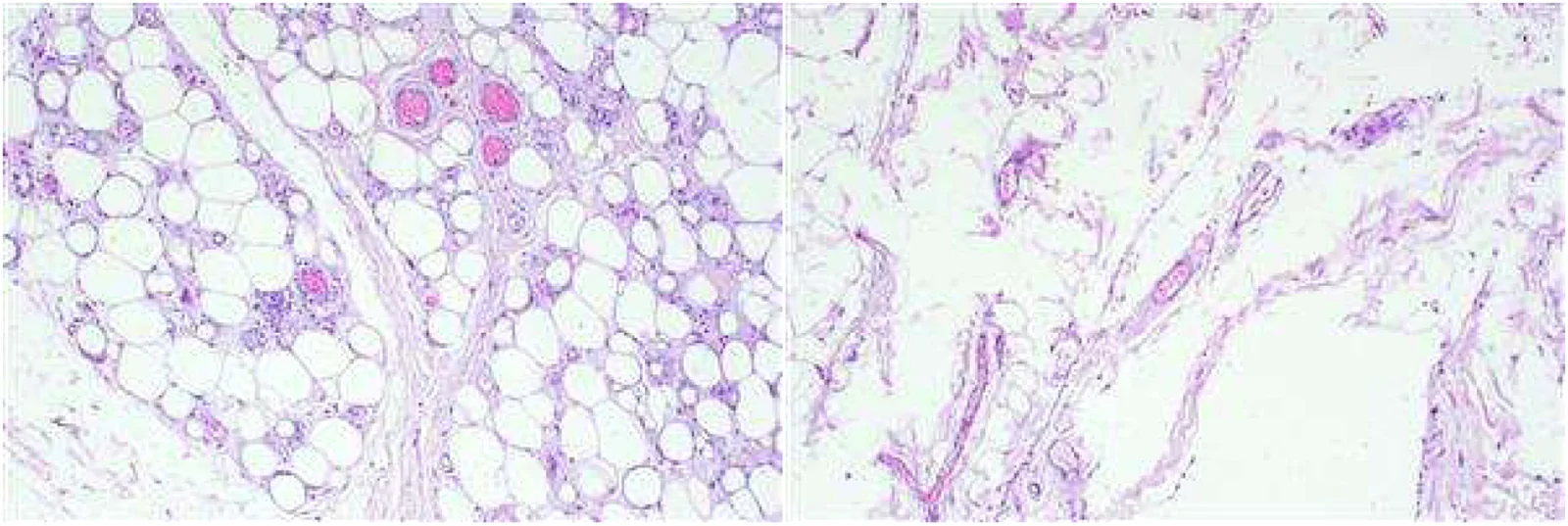
Pathological diagnosis: Fibrolipomatous vascular proliferative lesion (benign).

Gross examination revealed an irregular, pale-white firm tissue specimen. Histopathological examination with hematoxylin and eosin staining demonstrated dense fibrous tissue proliferation within a collagenous matrix, interspersed with mature adipocytes (no lipoblasts identified) and numerous thin-walled small blood vessels. No cellular atypia, nuclear pleomorphism, mitotic Fig.s, necrosis or inflammatory cell infiltration was observed (Fig. 1). The lesion was diagnosed as a benign fibrolipomatous vascular proliferative lesion.Histopathological examination of the right-sided specimen revealed normal mature adipose tissue without fibrosis, vascular proliferation, or cellular atypia.

Postoperative recovery was uneventful. The contour of the left lower eyelid improved significantly. At follow-up, the patient remained free of recurrence, with stable visual acuity, ocular motility and intraocular pressure.

## Discussion

This case illustrates a notable diagnostic pitfall: a deep orbital mass was completely masked by concomitant superficial orbital fat prolapse, leading to preoperative misdiagnosis. Although clinical diagnosis of typical OFP has high accuracy, orbital soft tissue masses with adipose components cover a wide differential diagnosis ranging from benign to malignant entities, and can be challenging to distinguish clinically.^6^^,^^7^ The superficial fat herniation in our patient presented classic features of OFP, giving no clinical indication for preoperative advanced imaging.

### Differential diagnosis

The differential diagnosis for lesions mimicking OFP includes multiple entities with distinct prognoses. Orbital lymphoma can present as medial fat pad prolapse with firm texture, and requires histopathological confirmation.^5^ Subconjunctival nerve sheath myxoma, a rare neoplasm, has also been misdiagnosed as OFP preoperatively.^4^ Other differential entities include orbital schwannoma, dermolipoma, lacrimal gland prolapse, liposarcoma and angiomyolipoma^.3,8^

Our case was diagnosed as a benign fibrolipomatous vascular proliferative lesion, characterized by proliferation of fibrous tissue, mature adipocytes and thin-walled small vessels. This histological pattern differs from angiomyolipoma (which contains smooth muscle and thick-walled vessels),^8^ and from simple reactive fibrovascular septa in OFP, as it forms a discrete mass with marked fibrous proliferation.^9^ The absence of atypia, mitosis and necrosis supports a benign nature, and the lesion may represent a hamartomatous or reactive proliferative process.

### Factors contributing to misdiagnosis

**Fig. 2:**
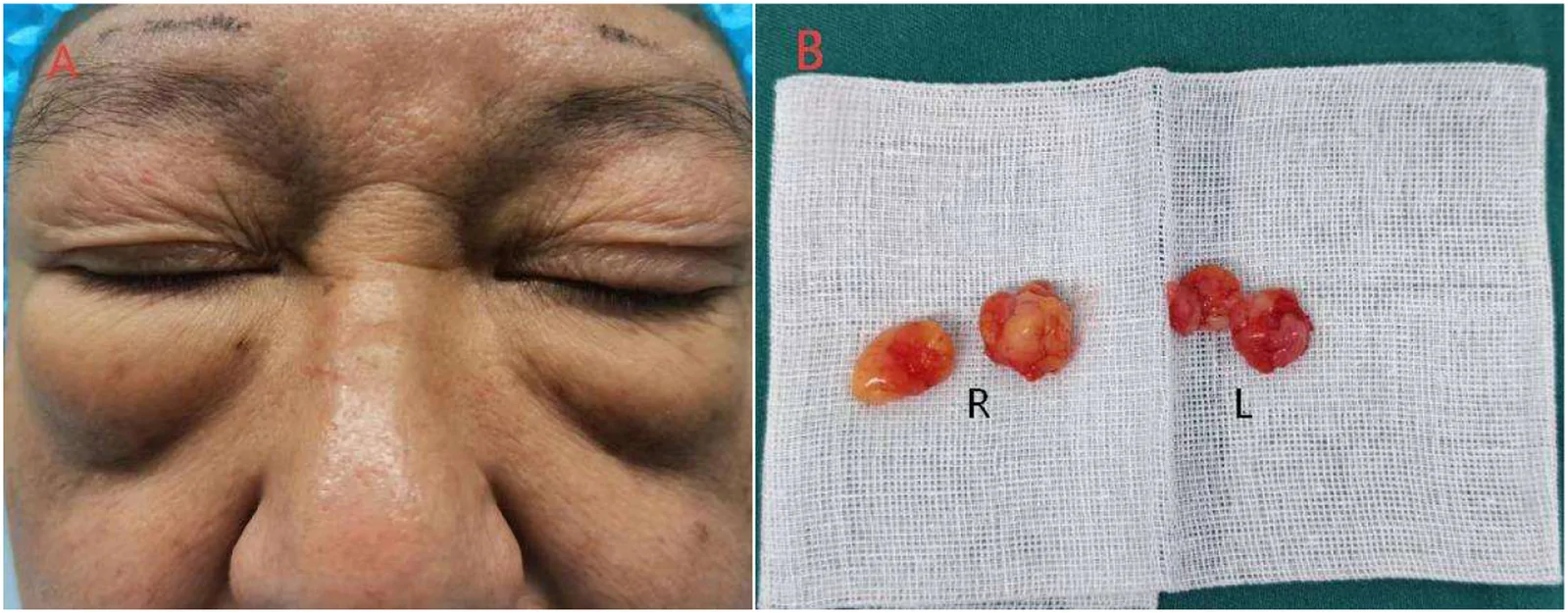
A: Preoperative view B: Comparative observation of excised adipose tissue intraoperatively.

Multiple factors led to the preoperative misdiagnosis in this case (Fig. 2). First, the patient’s age and clinical presentation were highly consistent with the typical epidemiological profile of OFP. Second, concurrent superficial fat herniation anatomically obscured the deep-seated mass. Third, the absence of red-flag symptoms (pain, visual decline, proptosis, motility restriction) provided no rationale for further imaging workup. Fourth, diagnostic anchoring bias clinicians tend to default to the most common diagnosis when findings align with a prevalent benign condition—reinforced the initial diagnosis^.10^

### Clinical implications and recommendations

Preoperative imaging (CT or MRI) is not routinely indicated for typical OFP, but should be considered for high-risk presentations: unilateral new-onset orbital fullness in elderly patients, atypical palpation findings (firm, non-reducible mass), rapid symptom progression, or concomitant neurological manifestations^11^ Advanced sequences such as diffusion-weighted imaging can further assist in differentiating benign from malignant lesions.

Intraoperatively, thorough exploration beyond superficial herniated fat is critical. When tissue characteristics (consistency, color, mobility) deviate from typical adipose tissue, complete excision and histopathological evaluation are warranted. As demonstrated in previous reports of lymphoma and rare tumors mimicking OFP, definitive diagnosis relies on histopathology, and all atypical excised orbital tissue should be submitted for histopathological examination.^4^^,^^5^

Based on our experience, we propose the following clinical recommendations: (1) Obtain detailed history on onset, progression and comorbidities for all presumed OFP cases; consider preoperative imaging for unilateral, new-onset or rapidly progressive lesions. (2) Perform careful intraoperative exploration beyond superficial fat, and proceed to complete excision for atypical tissue. (3) Submit all atypical excised orbital tissue for histopathological examination; normal orbital fat excised during routine, uncomplicated blepharoplasty need not be submitted unless there is clinical suspicion. (4) Maintain a broad differential diagnosis for apparent OFP. (5) Establish long-term follow-up for patients with unexpected pathological findings.

### Limitations

This is a single case report with inherent limitations in generalizability. The absence of preoperative imaging precludes characterization of the lesion’s radiographic features. Additionally, lack of immunohistochemical profiling limits more precise histopathological classification. Future cases should incorporate immunohistochemical panels to facilitate accurate nosological categorization.

In conclusion, orbital fat prolapse may coexist with or mask deeper orbital pathology. When intraoperative findings diverge from preoperative expectations, thorough exploration and routine histopathological examination are essential to avoid missed diagnoses. This case adds to the literature on orbital lesions mimicking common benign conditions, and highlights the value of intraoperative clinical judgment.

## Conclusions

We report an unusual case of a fibrolipomatous vascular proliferative lesion incidentally discovered during surgery for clinically diagnosed orbital fat prolapse in a 61-year-old male. The key clinical lessons derived from this case are:

1. Orbital fat prolapse may coexist with or mask deeper orbital pathology; a preoperative diagnosis of OFP should not preclude thorough intraoperative evaluation.
2. For unilateral, new-onset orbital fat prolapse in elderly patients, preoperative orbital imaging (CT or MRI) should be considered to exclude concurrent deep lesions.
3. When intraoperative tissue characteristics (consistency, color, depth) deviate from expected findings, aggressive exploration and complete excision for histopathological evaluation are warranted.
4. Histopathological examination of all atypical excised orbital tissue is essential.
5. This case adds to the growing body of literature documenting orbital lesions that mimic common benign conditions and highlights the critical importance of intraoperative judgment and pathological confirmation in ophthalmic surgery.

## Ethical considerations

### Ethics and consent to participate

This study was conducted in accordance with the Declaration of Helsinki. Written informed consent was obtained from the patient, and this retrospective case report was exempt from institutional ethical review.a copy of the signed consent form would be available on request for review by the Editor-in-Chief of this journal.

### Consent to publish

The patient provided written informed consent for publication of this report and all accompanying images, with all identifiable personal data fully anonymized.

## Availability of data and materials

All data supporting this study are included in the manuscript. Raw data are available from the corresponding authors upon reasonable request.

## Competing interest

The authors declare no competing interests.

## Funding

No funding was received for this work.

## Authors' contributions

P.Y. wrote the main manuscript and collected clinical data. M.B. and F.Z. and J.K. prepared all Fig.s and sorted pathological materials. X.L. and D.W. designed the study, performed the surgery and revised the manuscript. All authors reviewed and approved the final version of the paper.

All authors approved the final version of the manuscript.
